# A meta-analysis of public microarray data identifies gene regulatory pathways deregulated in peripheral blood mononuclear cells from individuals with Systemic Lupus Erythematosus compared to those without

**DOI:** 10.1186/s12920-016-0227-0

**Published:** 2016-11-15

**Authors:** Wendy Kröger, Darlington Mapiye, Jean-Baka Domelevo Entfellner, Nicki Tiffin

**Affiliations:** South African National Bioinformatics Institute/Medical Research Council of South Africa Bioinformatics Capacity Development Unit, University of the Western Cape, Cape Town, South Africa

**Keywords:** Lupus, Systemic Lupus Erythematosus (SLE), Microarray, Gene expression, Meta-analysis

## Abstract

**Background:**

Systemic Lupus Erythematosus (SLE) is a complex, multi-systemic, autoimmune disease for which the underlying aetiological mechanisms are poorly understood. The genetic and molecular processes underlying lupus have been extensively investigated using a variety of -omics approaches, including genome-wide association studies, candidate gene studies and microarray experiments of differential gene expression in lupus samples compared to controls.

**Methods:**

This study analyses a combination of existing microarray data sets to identify differentially regulated genetic pathways that are dysregulated in human peripheral blood mononuclear cells from SLE patients compared to unaffected controls. Two statistical approaches, quantile discretisation and scaling, are used to combine publicly available expression microarray datasets and perform a meta-analysis of differentially expressed genes.

**Results:**

Differentially expressed genes implicated in interferon signaling were identified by the meta-analysis, in agreement with the findings of the individual studies that generated the datasets used. In contrast to the individual studies, however, the meta-analysis and subsequent pathway analysis additionally highlighted TLR signaling, oxidative phosphorylation and diapedesis and adhesion regulatory networks as being differentially regulated in peripheral blood mononuclear cells (PBMCs) from SLE patients compared to controls.

**Conclusion:**

Our analysis demonstrates that it is possible to derive additional information from publicly available expression data using meta-analysis techniques, which is particularly relevant to research into rare diseases where sample numbers can be limiting.

**Electronic supplementary material:**

The online version of this article (doi:10.1186/s12920-016-0227-0) contains supplementary material, which is available to authorized users.

## Background

Systemic Lupus Erythematosus (SLE) is a multi-systemic autoimmune disease associated with high morbidity and mortality. At least nine out of ten lupus patients are female, with an onset age of between late teens and early forties. There is a significant difference in the prevalence of SLE occurring in different population and ethnic groups, and an occurrence of approximately 40 versus over 200 cases out of 100 000 persons among Northern European or black people respectively, has been reported [1, 2].

There is no predictable course for SLE, and patients can experience alternating periods of disease flare of varying severity; and remission, during which they have no obvious signs or symptoms. Lupus is also commonly misdiagnosed as it displays a broad range of clinical presentation resulting from inflammation and damage caused by the deposition of autoantibody-complexes in various tissues [3]. There is a wide variety of contributing factors attributed to the active disease phenotype and the precise pathological mechanisms of SLE have not yet been fully elucidated. Extensive work, however, has shown its aetiology to be multifactorial, with strong evidence indicating a substantial genetic component to SLE [4–9]. Lupus is now believed to be the result of a complex model in which multiple genes influence the likelihood of establishing the disease state in response to different environmental triggers [10]. The molecular basis underpinning this disease remains unclear, however, and more research is required to understand the mechanisms contributing to the lupus phenotype.

The genetic and molecular processes underlying lupus have been extensively investigated using a variety of -omics approaches, including genome-wide association studies, candidate gene studies and microarray experiments of differential gene expression in lupus samples compared to controls. Many of these data sets are in the public domain, and can be accessed freely by researchers [11–31]. These studies produce extensive and information-rich data sets that represent a snapshot of all genetic and/or molecular events occurring in a diseased cell at one particular point in time, and can be used to generate hypotheses on the molecular mechanisms underlying lupus.

The comparison and meta-analysis of such clinical datasets can be complicated by a variety of factors. Firstly, and especially with rare diseases such as SLE, sample size can be limiting both in the number of samples included in datasets and the number datasets within the public domain. Secondly, because participants in the study are usually in clinical care, it is often not possible to stratify cohorts by treatment regimes, particular clinical symptoms or participant demographics, and matched controls are often not available. Thirdly, the study design, participant inclusion criteria and sample type analysed are generally not standardised across studies. The wealth of information contained within these data sets is frequently underestimated, and often under-utilised once initial analyses have been completed.

In this study, we have aimed to address some of these factors by using an approach that focuses on identifying mechanistic pathways that may underlie SLE aetiology, rather than focusing on the identification of key individual genes. The benefits of addressing differential changes at a pathway level as opposed to a gene level have previously been described [32, 33]. We have analysed existing microarray data sets to identify regulatory networks and pathways that are dysregulated in SLE, including pathways that were not identified in the original individual studies. This is achieved using statistical approaches for meta-analysis of the combined results; using data from a variety of microarray expression experiments performed using different microarray platforms. We have conducted the analysis on the premise that not all genes in an aetiological pathway will necessarily show high levels of differential regulation; but that many genes of that pathway will be differentially regulated to some significant and detectable level. Our approach, therefore, uses less stringent criteria to select the differentially expressed gene lists followed by further more stringent pathway analysis. The first statistical approach to select differentially expressed genes is a binning method using quantile discretisation, and can analyse microarray datasets from different platforms; and the second independent method used to corroborate these results uses a classical scaled approach.

Our analysis demonstrates that it is possible to derive additional aetiological pathway information from publicly available expression data, using these meta-analysis techniques. This is particularly relevant to research into rare diseases where datasets tend to be fairly small, potentially limiting the statistical significance of findings from individual studies. Furthermore, this study highlights the value of sharing datasets in the public domain once primary analyses are completed.

## Methods

### Inclusion-exclusion criteria for datasets

Microarray studies investigating human peripheral blood mononuclear cells (PBMCs) or any subpopulation thereof (lymphocytes, monocytes or granulocytes) in at least four patients with SLE over the age of 16 were considered. SLE diagnosis satisfied the criteria set by the American College of Rheumatology [34] or similar. As we were particularly interested in genes differentially expressed during disease flare, lupus patients with disease activity scores of SLE Disease Activity Index (SLEDAI; [35, 36]) less than 6, or British Isles Lupus Assessment Group (BILAG) C, D or E [37, 38] were excluded. Patients on maintenance immunosuppressive treatment but still exhibiting an active disease phenotype (SLEDAI ≥ 6 and/or BILAG A or B) were included in the study. Samples that were cultured in any way after collection were excluded. Where only subsets of participants satisfying these criteria were identified, the data for these individuals were included in the analysis.

### Data collection and pre-processing

The ArrayExpress database [29] was used to identify microarray studies investigating participants with SLE compared to healthy controls. The search term used was “systemic lupus” and results were filtered by organism (Homo sapiens) and experiment type (RNA array assay), in April 2013.

Raw data for Affymetrix data sets [GEO:GSE11909] [16], [GEO:GSE13887] [17] and [GEO:GSE38351] [39] were obtained from Gene Expression Omnibus (GEO; [40]) using the R package GEOquery [41]. Samples fulfilling the specified criteria were Robust-Multi array Average normalised (Irizarry et al., 2003) using the simpleaffy R package [42, 43]. Another study using a custom spotted oligonucleotide array, [ArrayExpress:E-MTAB-145] [26], was also included. These raw data were imported into R using the ArrayExpress package [44], and the Limma package [45] was used for both print-tip loess within-array and quantile between-array normalisations of samples fulfilling the above criteria from E-MTAB-145.

Microarray probe identifiers (IDs) were converted to Ensemble IDs with a Python script accessing the Ensembl MySQL database (Ensembl 74: December 2013; [46]). Probe redundancy within each data set was resolved by averaging expression values for probe sets mapping to common Ensembl IDs and technical replicates were averaged, using a Python script.

### Meta- and differential expression analysis of microarray studies

Common Ensembl IDs between data sets were normalised across data sets using two different methods. As the aim of this study was to identify mechanistic pathways involved in lupus aetiology as opposed to individual genes, we used a less stringent approach in the selection of genes so as to provide a larger gene set for the pathway analysis in which we applied more stringent selection criteria.

The first recently developed method for the meta-analysis of micro-array data, a binning approach, was quantile discretization [47]. This method directly integrates disparate microarray data at the gene expression level and was first described to assess the benefit of performing supervised classification across disparate sources of microarray data [48]. In this study, we optimised this method to perform statistical differential expression analysis with an optimum quantile discretisation range of 128.

In the second method a scaling approach was employed. We centered and scaled the expression values corresponding to each sample. The result of this operation was to get a mean equal to 0 and a sample standard deviation equal to 1 for each sample (across all genes), which tends to cancel out the inter-sample variations that are non significant for the purpose of our study on the differential expression of genes between cases and controls.

Significantly differentially expressed genes were identified using the Wilcoxon Rank Sum Test [49] with Benjamini-Hochberg correction for multiple testing [50]. While we were able to apply a more stringent filter of *p*-value < 0.05 to the scaling dataset, we needed to relax this filter for the binning method to < 0.1 as all of the adjusted *p*-values obtained from this method were greater than 0.05.

### Pathway and regulatory network analysis

Differentially expressed genes between SLE patients in disease flare and normal controls identified through the binning and scaling methods were investigated using Ingenuity Pathway Analysis (IPA; [51]). The IPA methodology compares proportional representation of genes from a defined test set in a canonical pathway (a known, well-characterised pathway), compared to the proportional representation of the pathway genes in the entire set of known genes. The *p*-value is calculated using a right-tailed Fisher Exact test and indicates the likelihood of the pathway association under the random model. The adjusted Benjamini-Hochberg *p*-value for a 5% FDR was calculated as 1.51 × 10^−3^ for the binning method and 2.3 × 10^−3^ for the scaling method, and controls for errors in selecting canonical pathways from a large set of options. The most over-represented canonical pathways enriched with differentially regulated genes were identified and an analysis of regulatory networks was undertaken. This was used to identify key common upstream transcription factors that may be driving cascades of differential gene regulation, as well as to identify any key node genes that might be crucial to the differential regulation of gene regulatory pathways and networks in the disease state.

## Results

### Data collection and normalisation across studies

**Fig. 1:**
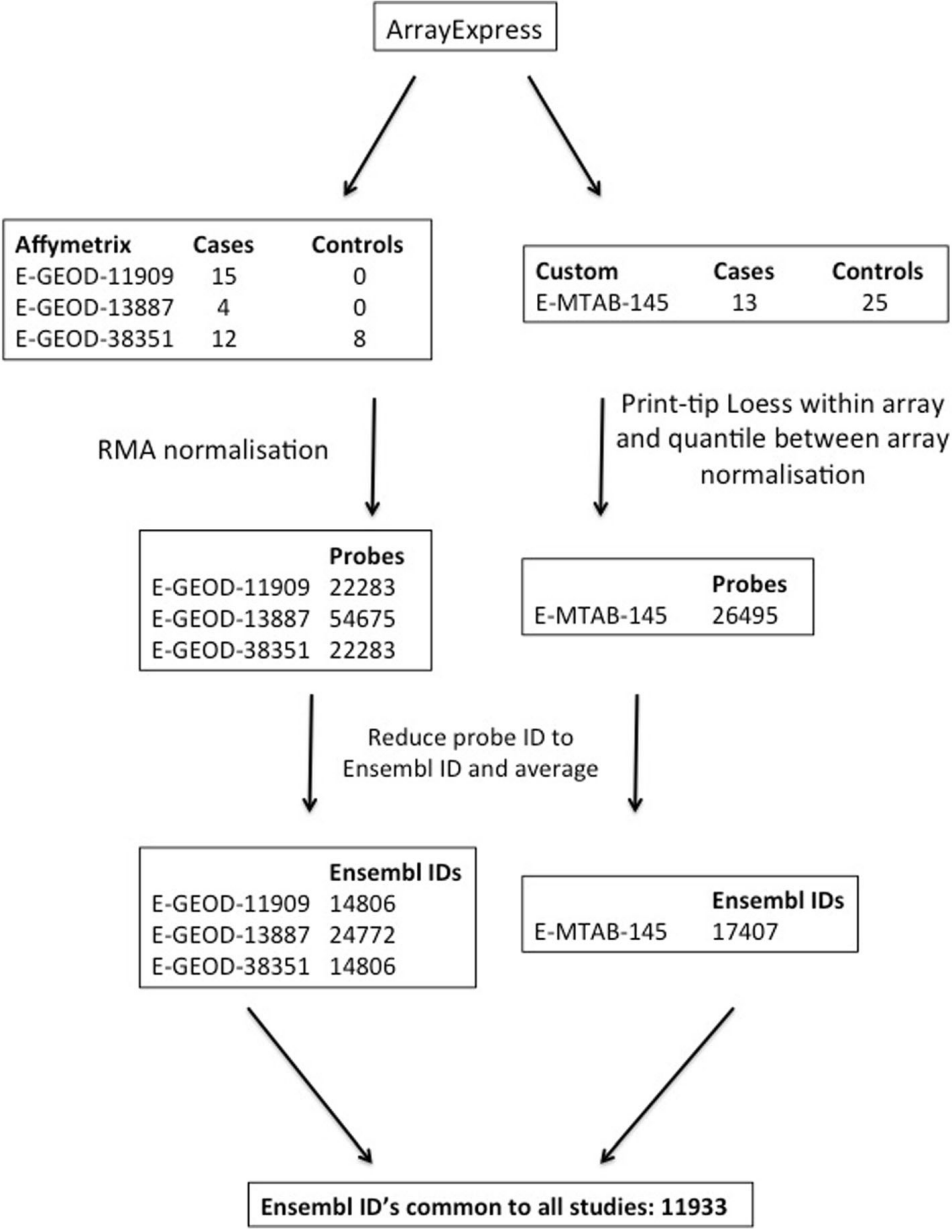
Data processing methodology. Summary of processing of data sets to generate a list of common gene expression matrices for each study

Four studies fulfilling the inclusion-exclusion criteria were selected for this meta-analysis study (Fig. 1). GSE11909 contributed 15 cases (no controls) from PBMCs [16], GSE13887 [17] contributed 4 cases (no controls) from CD3-positive T cells [39], GSE38351 contributed 12 cases and 8 controls from monocytes, and E-MTAB-145 contributed 13 cases and 25 controls from PBMCs (Fig. 1, [26]). Normalised data sets GSE11909, GSE13887, GSE38351, E-MTAB-145 each contained 22,283, 54,675, 22,283 and 26,495 probes respectively. These probe sets were reduced to 14,806, 24,772, 14,806 and 17,407 genes (respectively), of which 11,933 genes (Ensembl ID) were common across all three data sets (Fig. 1).

### Meta- and differential expression analysis of microarray studies

Two approaches were used to normalise the expression values across data sets. The binning and scaling methods resulted in lists of 749 (*p*-value < 0.1) and 597 (*p*-value < 0.05) differentially expressed genes respectively [see Additional file 1], with 458 of these genes common between the lists. We took the decision to use relatively relaxed *p*-value cutoffs for differential expression analysis in light of the fact that we were interested in identifying mechanistic pathways underlying lupus, rather than identifying individual key genes, and that we were using more stringent criteria in the pathway analysis component of the study.

### Pathway and regulatory network analysis

The top overlapping canonical pathways identified by IPA using both binning and scaling gene lists were *Agranulocyte Adhesion and Diapedesis* and the *Role of Pattern Recognition Receptors in Recognition of Bacteria and Viruses*; both with *p*-values < 2 × 10^−04^ (Table 1). Other pathways identified included *Interferon Signaling*, *Oxidative Phosphorylation* and *Toll-like Receptor Signaling* from the binning method (*p*-value < 7 × 10^−04^), and the *Role of Cytokines in Mediating Communication between Immune Cells*, the *Role of Hypercytokinemia/hyperchemokinemia in the Pathogenesis of Influenza* and *Role of Macrophages* and *Fibroblasts and Endothelial Cells in Rheumatoid Arthritis* for the scaling method (*p*-value < 2 × 10^−04^). Genes assigned to the pathways are shown in Table 2. When using Benjamini-Hochberg adjusted *p*-values, the top five pathways from either method are significant within an FDR of 5%.

**Table 1:** Top canonical pathways enriched for differentially regulated genes. Top Canonical Pathways identified for the gene lists identified through the Binning and Scaling methods of normalisation across studies

Top Canonical Pathway	Binning method		Scaling method	
	*p*-value	Ratio	*p*-value	Ratio
Agranulocyte Adhesion and Diapedesis	1.59 × 10^-05 §^	19/190 (0.1)^§^	1.37 × 10^-04 §^	15/190 (0.079)^§^
Role of Pattern Recognition Receptors in Recognition of Bacteria and Viruses	7.11 × 10^-05 §^	14/127 (0.11)^§^	1.18 × 10^-04 §^	12/127 (0.094)^§^
Role of Cytokines in Mediating Communication between Immune Cells	2.23 × 10^−03^	(0.13)	1.27 × 10^-05 §^	9/56 (0.161)^§^
Role of Hypercytokinemia/Hyperchemokinemia in the Pathogenesis of Influenza	3.24 × 10^−03^	(0.13)	1.8 × 10^-05 §^	8/45 (0.178)^§^
Interferon Signaling	9.58 × 10^-05 §^	7/34 (0.206) ^§^	2.14 × 10^−04^	0.18
Oxidative Phosphorylation	1.4 × 10^-04 §^	13/119 (0.109) ^§^	0.197	0.042
Role of Macrophages, Fibroblasts and Endothelial Cells in Rheumatoid Arthritis	1.10 × 10^−03^	0.07	1.45 × 10^-04 §^	20/304 (0.066)^§^
Toll-like Receptor Signaling	6.67 × 10^-04 §^	9/74 (0.122) ^§^	3.02 × 10^−03^	0.095

^§^indicates the top five pathways for each method; the ratio represents the number of involved genes divided by the total number of genes in the pathway; the *p*-value indicates over-representation of genes in the pathway

**Table 2:** Genes implicated in the top canonical pathways (HUGO gene symbols)

Pathway	Binning Method	Scaling Method
Agranulocyte Adhesion and Diapedesis	ICAM2,PF4, MYH1, CLDN4, HRH1, MYH10, CCL1, CLDN1, IL37, CCL19, ITGA1, IL1A, AOC3, MYH8, CCL17, IL36G, MMP1, MYH13, MMP16	ICAM2, PF4, MYH1, MYH2, CLDN4, CCL1, CLDN1, IL37, CCL19, ITGA1, MYH8, IL36G, MYH13, IL33, MMP16
Role of Pattern Recognition Receptors in Recognition of Bacteria and Viruses	OAS1, IL3, NOD1, OAS2, DDX58, NFKB2, IL11, TLR3, IFNA1/IFNA13, OAS3, IL1A, IL5, CNTF, PRKD1	MBL2, OAS1, IL2, IL3, IL11, TLR3, IFNA1/IFNA13, OAS3, OAS2, IL12B, DDX58, PRKD1
Role of Cytokines in Mediating Communication between Immune Cells	IL3, IL15, IFNA1/IFNA13, IL1A, IL5, IL36G, IL37	IL2, IL3, IL15, IFNA1/IFNA13, IL12B, IL36G, IFNA2, IL33, IL37
Role of Hypercytokinemia/Hyperchemokinemia in the Pathogenesis of Influenza	IL15, CCR1, IFNA1/IFNA13, IL1A, IL36G, IL37	IL15, CCR1, IFNA1/IFNA13, IL12B, IL36G, IFNA2, IL33, IL37
Interferon Signaling	OAS1, IRF9, IFIT1, IFNA1/IFNA13, IFIT3, IFITM2, MX1	OAS1, IFIT1, IFNA1/IFNA13, IFIT3, IFITM2, MX1
Oxidative Phosphorylation	CYCS, UQCR11, NDUFS1, COX6B1, ATP5G2, NDUFA9, NDUFA7, COX6A1, NDUFS5, ATP5H, NDUFB1, ATP5C1, ATPAF2	COX6B1, NDUFA7, COX6A1, ATPAF2, NDUFS1
Role of Macrophages, Fibroblasts and Endothelial Cells in Rheumatoid Arthritis	IRAK1, IL15, WNT7B, CHP1, SFRP1, DKK1, TRADD, FZD7, IL37, FZD5, IRAK4, WNT2B, PRSS1, TLR3, CCND1, IL1A, PRKD1, WIF1, IL36G, MMP1, FCGR3A/FCGR3B	IL15, TNFSF11, WNT7B, CHP1, DKK2, SFRP1, DKK1, TRADD, FZD7, IL37, FZD5, IRAK4, PRSS1, TLR3, CCND1, PRKD1, WIF1, IL36G, IL33, FCGR3A/FCGR3B
Toll-like Receptor Signaling	NFKB2, IRAK1, TNFAIP3, TLR3, IL1A, TAB2, IL36G, IL37, IRAK4	TNFAIP3, TLR3, IL12B, IL36G, IL33, IL37, IRAK4

From these pathways, IPA calculated the top overlapping upstream regulators to be IFNL1, IFNA2, TNF and IRF7; all with *p*-values < 2 × 10^−13^ (Table 3). Tretinoin and IRF3 were additionally identified from the binning and scaling methods, respectively.

**Table 3:** Top upstream regulators for differentially regulated genes. Top upstream regulators, shown by HUGO gene symbols, identified by IPA for the gene lists identified through the Binning and Scaling methods of normalisation across studies

Top Upstream Regulators	*p*-value	
	Binning method	Scaling method
IFNL1	2.20 × 10^−21^	4.06 × 10^−21^
IFNA2	1.87 × 10^−20^	3.13 × 10^−18^
Tretinoin	2.55 × 10^−17^	1.70 × 10^-11 ¥^
TNF	4.33 × 10^−14^	8.91 × 10^−13^
IRF7	5.85 × 10^−14^	1.40 × 10^−13^
IRF3	1.99 × 10^-8 ¥^	1.24 × 10^−12^

^¥^indicates *p*-values that do not fall in the top five

## Discussion

In this study, we have used publicly available microarray datasets to detect genes that are differentially regulated in blood cells from people with SLE when compared to controls without SLE. By combining the data from four separate gene expression studies in a meta-analysis, we hoped to be able to derive additional information from the data that possibly could not be established from the individual studies in isolation. In general, we found this to be the case, with confirmation of the previously reported findings of the separate studies; as well as some additional insights that were not reported in the individual papers. Despite the lack of control samples from two of the studies, the results of the meta-analysis do not seem to be biased towards the studies which included controls, as evidenced by identification of *Toll-like Receptor Signaling* and *Oxidative Phosphorylation* which were not identified in those original individual studies that contained controls. Although the ideal scenario would be an equal number of cases and controls where possible, using the combined meta-analysis approach and increasing the number of datasets for cases can still lead to detecting smaller effect sizes with accuracy even in the absence of a concomitant increase in number of control samples. A potential source of bias in this meta-analysis is the lack of controls for two of the studies, and because the study analyses previously published data this lack of controls for two studies cannot be addressed retrospectively. Whilst the meta-analysis including all studies increases sample size and statistical power, and the analysis is designed to control for false positives, we acknowledge that it is not possible to completely rule out the possibility of such bias. In particular, we focused our analysis on identifying aetiological pathways rather than specific aetiological genes, with inclusive criteria when selecting differentially regulated genes. The rationale behind this decision was two-fold: firstly, we aimed to select pathways for which a large proportion of genes are differentially regulated in preference to pathways where fewer genes are consistently subject to large fold changes in expression; and secondly, we aimed to accommodate the different characteristics and small sample sizes of the datasets used.

**Fig. 2:**
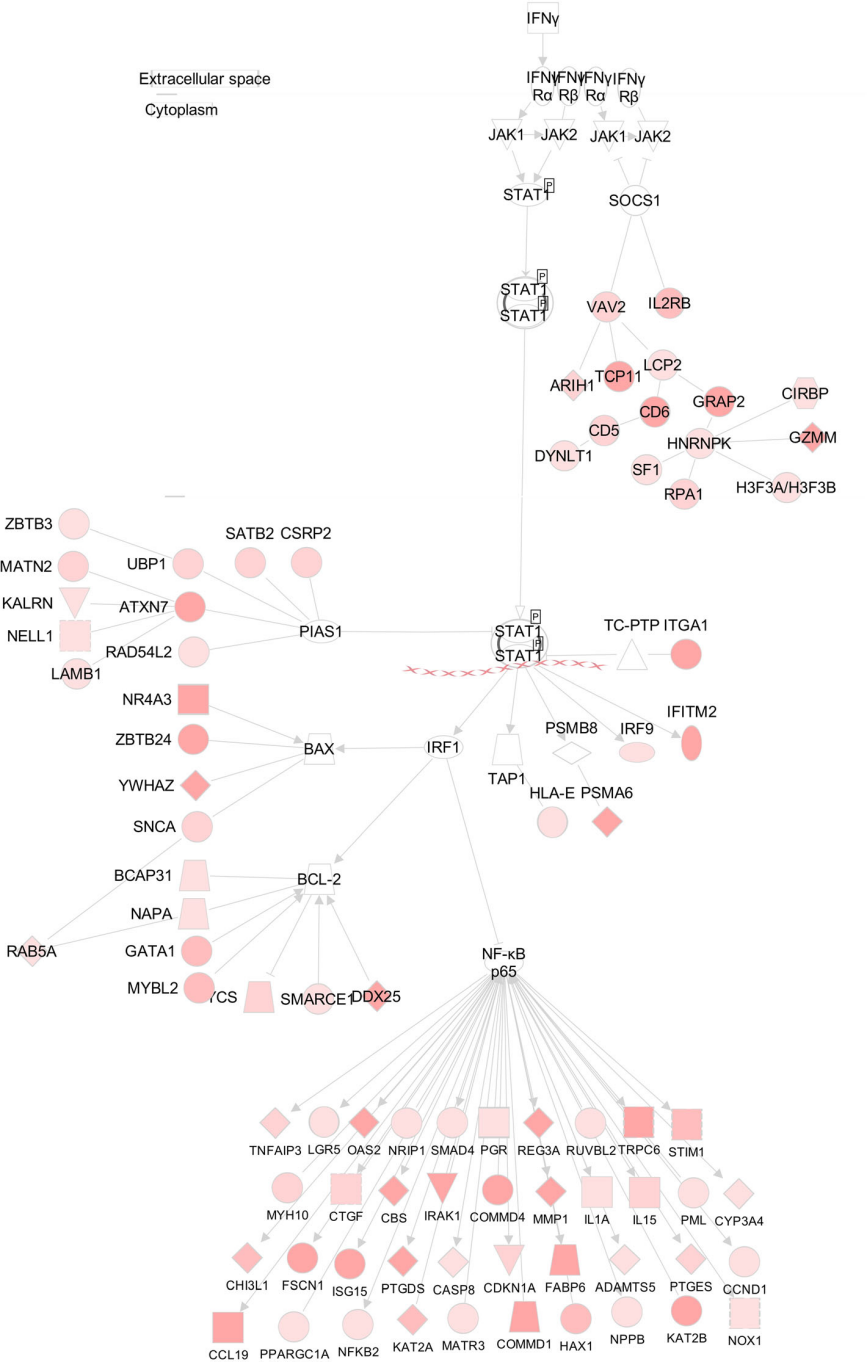
Lupus interferon model. Proposed model for differentially expressed genes found in lupus patients that are either integral to interferon signaling or factors that lie directly downstream of interferon pathways

Activation of the interferon (IFN) pathway in lupus patients is well established [52], and is the common underlying theme found in all of the original individual studies used in this meta-analysis [16, 17, 26, 39], as well as in other similar meta-analysis studies of lupus data [53, 54]. In this meta-analysis, we similarly found that when using both the binning and scaling methods, IFN signaling was a top canonical pathway activated in lupus patients compared to controls (Table 1), providing a good positive control for our meta-analysis, and corroborating the findings of the individual analyses. Differentially regulated genes that are either integral to IFN signaling or lie directly downstream to the IFN pathways are shown in Fig. 2.

The IFN family of signaling proteins is a subset of cytokines with a protective function elicited in response to pathogenic species, such as bacteria or viruses. In lupus patients, type 1 IFN signaling stimulates persistent dendritic cell activation and has direct effects on B and T cell activation. Dendritic cells are able to selectively activate autoreactive T cells, while activated B cells seem to play a role in elevated autoantibody production and immune complex manifestation [55]. The presence of pathogens also stimulates elevation of other cytokines that signal immune cells to migrate to places of infection. These activated immune cells are in turn stimulated to produce more cytokines thereby creating a positive feedback loop. In normal healthy individuals, this process is tightly controlled. However deregulation of this control, sometimes present in patients with rheumatic diseases, can lead to cytokine storm, or *hypercytokinemia*: a potentially fatal complication that can lead to severe tissue and organ damage. Interestingly, while hypercytokinemia is quite rarely found in adult lupus patients [56], it has been more commonly reported in juveniles with this disease [57]. While our selection criteria only included individuals of over 16 years of age, the study by Chaussabel et al., was in fact on a group of pediatric lupus patients, of which we included 15 individuals over 16 years in our meta-analysis [16]. This may have enriched the set of differentially regulated genes that we identified here for IFN signaling events that are implicated in hypercytokinemia.

In addition, however, our study of the combined data identified a number of other differentially regulated pathways in lupus patients compared to controls.

Of particular interest, was the detection of activated *toll-like receptor* (TLR) signaling (Table 1) and the *role of pattern recognition receptors* (PRRs) *in recognition of bacteria and viruses*. Importantly, the latter pathway was a similar result to Makashir et al.’s findings of enrichment of genes within the module of immune defense against extracellular organisms [54]. This highlights the benefits of using multiple methodologies to explore existing datasets, as different analytical approaches may bring a variety of evidences together towards the same conclusions. The TLR class of proteins, a subset of PRRs, has an essential role in the mammalian innate immune response [58]. Along with other PRRs, these proteins are able to recognise structurally conserved microbial molecular patterns, also known as pathogen-associated molecular patterns (PAMPs). Activation of TLRs leads to a complex response which involves activation of IFN signaling and can have downstream roles in both apoptosis and autophagy [59]. While none of the original studies highlighted a change in this pathway for any of the naive patient samples, Smiljanovic et al. (2012) report TLR2 to be up-regulated in their tumor necrosis factor (TNF)-α in vitro-treated lupus monocytes compared to controls [39]. Furthermore, polymorphisms in TLR3, TLR7 and TLR9 genes have been associated with lupus patients in some populations [60–62] and a number of in vivo and in vitro studies have strongly implicated a number of TLRs to play a role in the pathogenesis of lupus [58]. By combining the datasets from the individual microarray studies here, we have similarly identified TLR signaling as a key regulatory mechanism that is differentially regulated in the PBMCs from lupus patients when compared to unaffected controls; and have demonstrated consistent differential regulation of TLR3.

*Oxidative phosphorylation* was another key pathway highlighted in our meta-analysis. This is a metabolic process through which ATP is produced by the release of energy from a series of redox reactions. These reactions involve the transfer of electrons between donor and acceptor pairs, performed by a group of protein complexes situated within the mitochondrial membrane. Simultaneously, this electron transport chain is coupled with the transport of protons across the membrane, setting up an electrochemical gradient within the inter-membrane space. ATP synthase is then able to exploit this gradient through chemiosmosis, allowing the phosphorylation of ADP, to produce ATP. Gene expression analysis by Fernandez et al., (2009) found increases in mitochondrial mass and membrane potential, as well as enhanced levels of NO production and intracellular calcium in lupus samples compared to controls. They also showed that the mammalian target of rapamycin (mTOR) activity is increased in this disease. mTOR is situated on the external mitochondrial membrane, and plays an essential role in the oxidative capacity of mitochondria, and pharmacological inhibition of mTOR by rapamycin has been shown to reduce the ATP generating capacity of mitochondria [63, 64]. Moreover, oxidative stress is known to be increased in lupus patients compared to healthy controls [65], and a number of oxidative stresses have been shown to influence oxidative phosphorylation [66, 67]. Taken together, it is likely that there will be an intimate interplay of changes in lupus patients between these processes and oxidative phosphorylation. Our meta-analysis has highlighted components of this pathway as being differentially regulated in PBMCs from lupus patients, suggesting that further research to explore a role for oxidative phosphorylation in SLE pathogenesis is warranted.

*Diapedesis*, or leukocyte extravasation, is the movement of white blood cells out of the vascular system via intact vessel walls into adjacent inflammation-affected tissue. As inflammation is a highly common disease manifestation found in lupus patients, it is reasonable to assume that diapedesis signaling will be markedly deregulated in lupus patients. This is further supported by the findings of Makashir et al., 2015 reporting an enrichment of genes involved in wounding and leukocyte cell migration modules within lupus patients, and importantly, both studies similarly identified FCGR3B as a key player within the associated pathway [54]. Together with ITGB2, ITGAM forms the Macrophage-1 antigen (Mac-1) integrin or complement receptor 3 (CR3), known to be involved in leukocyte extravasation and phagocytosis. Notably, a number of polymorphisms within or near the gene encoding Integrin alpha M, or ITGAM, have been highly associated with genetic lupus risk factors, with the *rs1143679* variant resulting in an R77H substitution in its gene product [68–71]. Interestingly, cells transfected with this mutant showed reduced ligand (ICAM-1 and ICAM-2) and complement (iC3b) binding abilities well as impaired iC3b-mediated phagocytosis, compared to those transfected with wild type ITGAM [72]. Rhodes and co-workers confirmed most of these results in ex vivo monocytes (adhesion) and macrophages (phagocytosis) from wild type or R77H homozygous volunteers, and also reported reduced adhesion to fibrinogen and DC-SIGN, the dendritic cell-specific intercellular adhesion molecule-3Grabbing non-integrin receptor, in R77H homozygous monocytes [73]. More recently, Fossati-Jimack and co-workers provided strong evidence suggesting that this variant’s role in lupus susceptibility is most likely due to its effects on clearance of cellular debris. They were only able to corroborate findings of reduced iC3b-directed phagocytosis in monocytes, macrophages, neutrophils and dendritic cells heterozygous for the R77H/R allele; the genotype most commonly associated with lupus disease [74]. The dysregulation of these key functions of diapedesis and adhesion, detected in our study through differential expression of key molecules in PBMCs from SLE patients, may therefore be contributing to pathogenic mechanisms in SLE.

From the gene lists in Table 2, it can be seen that there is frequent overlap in gene membership between the different pathways identified. This reflects the strong interplay that exists between the pathway functions and regulatory mechanisms that drive dysregulated cellular mechanisms; and is consistent with the complexity of SLE aetiology whereby multiple downstream effects of gene dysregulation can result in the complex disease phenotype. Identification of common upstream regulators of these pathways, however, may provide insights into key molecules that are fundamental to the dysregulated cellular processes underlying SLE aetiology.

IFN-lambda 1 (IFNL1, also known as IL29), a type III IFN, has been proposed previously as a key molecule in renal disorder and arthritic progression in SLE [75]*.* The role of IFN alpha in SLE has been extensively explored (reviewed in [76]), and IFN-alpha 2 (IFNA2) has recently been implicated in perpetuation of SLE disease activity [77]. Furthermore, a number of members highlighted within the IFN pathway in this study have previously been reported in other meta-analyses, including IFIT1, IFIT3, MX1, IRF7 and OAS1 [53, 54]. TNF is already targeted by biologic therapeutic approaches [78], and IFN regulatory factors 3 and 7 (IRF3 and IRF7) have been implicated in the IFN signature in lupus previously [54, 79, 80]. Whilst pathway analysis highlighted tretinoin as a common upstream regulator of identified pathways, there is little information about a role for tretinoin, belonging to the family of retinoids, in SLE. Whilst some studies describe the use of retinoids for topical treatment of cutaneous lupus, for example [81]; and a case study describes the use of retinoids in treating patients with lupus nephritis [82]; any relationship that may exist between this molecule and SLE progression is, as yet, unclear.

It is important to remember that the cellular composition of PBMCs can change in the lupus disease state, with increases in myeloid and decreases in lymphoid lineages observed in lupus PBMCs compared to control samples. These changes can also bring about differences observed in gene regulation [26], depending on the cell population that is assayed. With this in mind, we are aware that the inclusion of specific monocyte and T cell subsets within our PBMC meta-analysis may bias the end result because of exclusion of the other cell types in generating these datasets. We think it is likely, however, that this bias would be most likely to dilute the observed differential expression between disease state and controls; rather than enriching a signal from the selected cell types. If this were the case, this would tend to result in concealment of a gene signature of interest, rather than indicating false positive differential regulation that does not occur in all datasets.

## Conclusions

This study demonstrates the value that can be gained from using appropriate statistical methods to combine expression datasets from multiple studies that compare gene expression between PBMCs from SLE patients compared to controls. The methods used here are robust even across different microarray platforms, and the pathways that are enriched for differentially regulated gene expression are consistent with the primary findings of the individual studies. Furthermore, the meta-analysis of multiple datasets has detected gene expression signatures additional to those described by the individual studies; and these regulatory pathways have been implicated in SLE through independent research approaches, confirming the validity of this meta-analysis. It is likely that the knowledgebase of regulatory pathways has been expanded since the individual studies were undertaken, and this may contribute in part to the increased range of regulatory mechanisms that were identified by the current meta-analysis. This further indicates the value in revisiting earlier datasets to extract more information about molecular processes underlying the disease state.

Such approaches may have particular value in the study of rare diseases, where study size can be limiting; and also where available datasets are clinically heterogeneous and cannot be stratified by stringent clinical or sampling criteria. Whilst bioinformatics analyses of existing datasets are currently unlikely to provide unequivocal clinical answers to disease aetiology, we believe that expanding the range of methodologies to explore such datasets can identify new mechanistic pathways to explore in ongoing and future research. Such studies highlight the utility of controlled public access to existing datasets, where ethical requirements for data re-use can be met, in order to increase our understanding of molecular mechanisms contributing to the aetiology of rare diseases.

## Additional file

Additional file 1: FDR-corrected *p*-values. (XLS 110 kb)

## Abbreviations

GEO: Gene Expression Omnibus
ID: Identifier
IFN: Interferon
IFNA: Interferon-alpha
IFNL: Interferon-lambda
IPA: Ingenuity pathway analysis
IRF: Interferon regulatory factors
mTOR: Mammalian target of rapamycin
PBMCs: Peripheral blood mononuclear cells
PRR: Pattern recognition receptors
SLE: Systemic Lupus Erythematosus
TLR: Toll-like receptor
TNF: Tumor necrosis factor
